# Supplementary material for: COZOID: contact zone identifier for visual analysis of protein-protein interactions
Source: BMC Bioinformatics. 2018 Apr 6;19:125. doi: 10.1186/s12859-018-2113-6 (PMC5889581; doi:10.1186/s12859-018-2113-6)
Supplement: Supplementary file 2 — Software build. Executable binary file of the software tool. (ZIP 118,784 kb) [file 12859_2018_2113_MOESM2_ESM.zip › cozoid/java/docs/beansbinding-1.2.1-doc.zip!/deprecated-list.html]

Deprecated List (Beans Binding)


---


|  |  |  |
| --- | --- | --- |
| **Overview** \| Package \| Class \| Use \| **Tree** \| **Deprecated** \| **Index** \| **Help** | |  |
| PREV   NEXT | **FRAMES**    **NO FRAMES**     **All Classes** |  |


---


## **Deprecated API**


---

**Contents**

- Deprecated Methods

**Deprecated Methods**

org.jdesktop.el.impl.parser.SimpleCharStream.getColumn()

org.jdesktop.el.impl.parser.SimpleCharStream.getLine()

org.jdesktop.beansbinding.AbstractBindingListener.sourceEdited(Binding)
  
          *This method has been replaced by `AbstractBindingListener.sourceChanged(org.jdesktop.beansbinding.Binding, org.jdesktop.beansbinding.PropertyStateEvent)` and it
will go away soon. It is being kept for a short period only,
to assist in migration.*

org.jdesktop.beansbinding.AbstractBindingListener.targetEdited(Binding)
  
          *This method has been replaced by `AbstractBindingListener.targetChanged(org.jdesktop.beansbinding.Binding, org.jdesktop.beansbinding.PropertyStateEvent)` and it
will go away soon. It is being kept for a short period only,
to assist in migration.*

---


|  |  |  |
| --- | --- | --- |
| **Overview** \| Package \| Class \| Use \| **Tree** \| **Deprecated** \| **Index** \| **Help** | |  |
| PREV   NEXT | **FRAMES**    **NO FRAMES**     **All Classes** |  |


---
